# *Trichophoromyia auraensis* is a putative vector

**DOI:** 10.1590/0074-02760170024

**Published:** 2017-07

**Authors:** Carolina Bioni Garcia Teles, Felipe Arley Costa Pessoa, Jansen Fernandes Medeiros, Luís Marcelo Aranha Camargo

**Affiliations:** 1 Fundação Oswaldo Cruz-Fiocruz, Porto Velho, RO, Brasil; 2 Universidade Federal de Rondônia, Porto Velho, RO, Brasil; 3 Fundação Oswaldo Cruz-Fiocruz, Instituto Leônidas e Maria Deane, Manaus, AM, Brasil; 4 Universidade de São Paulo, Instituto de Ciências Biomédicas, Monte Negro, RO, Brasil; 5 Faculdade São Lucas, Porto Velho, RO, Brasil; 6 Instituto Nacional de Epidemiologia na Amazônia Ocidental, Porto Velho, RO, Brasil

**Keywords:** Leishmania, disease vectors, Acre, Brazil, Trichophoromyia auraensis

## Abstract

The sandfly *Trichophoromyia auraensis* has recently evolved as a proven vector of *Leishmania (Viannia)* endemic to state of Acre in the north of Brazil. This note is intended to propose a correction in the report of the first occurrence of natural infection of *Leishmania (Viannia)* in this species. We and the other scientific groups reinforced that *Tr. auraensis* is a possible vector involved in the transmission of American cutaneous leishmaniasis in Acre, Brazil.

About the article: First description of *Leishmania* ( *Viannia* ) infection in *Trichophoromyia auraensis* (Psychodidae: Phlebotominae) in the transmission area of American cutaneous leishmaniasis in Acre state, Amazon Basin, Brazil.

American cutaneous leishmaniasis (ACL) is a disease highly endemic to the state of Acre, Brazil. This state is located on the Brazilian border and is surrounded by Peru and Bolivia, both of which have a high number of ACL cases reported. The state of Acre (Brazil) was the unit of the federation with the highest incidence of ACL in 2010, reaching 141.64 cases per 100,000 inhabitants. However, very few studies have been conducted on the development of sandfly ecology in this state. A quick search on Scopus demonstrated that only seven studies researching on this topic in the state of Acre have been published thus far. Valdivia et al. (2012) , who found *Trichophoromyia auraensis* (Mangabeira) infected with *Leishmania* ( *Viannia* ) in Madre Dios, the Peruvian state that borders Acre, indicated that it is the main vector of *Leishmania* ( *Viannia* ) in the Southwest Amazon Basin. In Acre, both the microregion of Rio Branco and a municipality (Assis Brasil) located in the microregion of Brasileia, are considered endemic areas of epidemiological relevance for ACL. According to the Notifiable Diseases Information System ( SINAN 2014 ), 387 cases were reported in the municipality between 2007 and 2013, corresponding to an annual mean detection coefficient of 93 ACL cases per 10,000 inhabitants.

Owing to its epidemiological importance, our research groups chose this area for conducting some epidemiological studies focusing on the ecological aspects of sandflies and *Leishmania* . In this region, 13 new occurrences were registered and two new species of sandflies were described ( Teles et al. 2013 , Oliveira et al. 2015 ). Moreover, the species of *Leishmania* in human cases were characterised by polymerase chain reaction (PCR) ( Teles et al. 2015 ).

In July 2016, our team published an epidemiological study conducted on almost seven thousand collected sandflies in Memórias do Instituto Oswaldo Cruz (MIOC) ( Teles et al. 2016 ). The females were grouped by pools and examined for the DNA tracks of *Leishmania* . We found that specimens of *Lutzomyia davisi* and *Lu. auraensis/Lu. ruifreitasi* contained the DNA tracks of *Leishmania* from the *L. braziliensis* complex at high concentrations. We registered this as the first report of the infection of *Lu. auraensis* / *Lu. ruifreitasi* by *L.* ( *V.* ) *guyanensis* and *L.* ( *V.* ) *braziliensis* in Brazil.

This note is to make a scientific correction; recently, in MIOC, January 2017, de Araujo-Pereira et al. (2017) assigned the first description of *Leishmania* ( *V.* ) infection in *Tr. auraensis* in the Rio Branco municipality of Acre state. The note of de Araujo-Pereira et al. (2017) , despite not inedited to the occurrence, is important because it reinforces that it is possible to find the infected populations of *Tr. auraensis* in some hundreds of kilometres of the endemic regions considered as ACL hot spots such as Assis Brasil, in Acre. This species also occurs in other Brazilian states such as Amazonas, Pará, Rondônia and Mato Grosso, which are characterised by high population, highways, intense deforestation, and intense migration caused by economical attractiveness. Therefore, these states should be studied more deeply to understand the role of this sandfly in the transmission of ACL.
