# Supplementary material for: Trichophoromyia auraensis is a putative vector
Source: Mem Inst Oswaldo Cruz. 2017 Jul;112(7):517–9. doi: 10.1590/0074-02760170024 (PMC5452491; doi:10.1590/0074-02760170024)
Supplement: Related texts

# Reply: Comments about the finding of *Trichophoromyia auraensis* (Mangabeira 1942) sandfly specimens infected by *Leishmania (Viannia)* parasites in Acre state

**Authors:** Thais de Araujo-Pereira, Daniela de Pita-Pereira, Mariana Côrtes Boité, Myllena Melo, Taiana Amancio da Costa-Rego, Andressa Alencastre Fuzari, Reginaldo Peçanha Brazil, Constança Britto

**Affiliations:** Laboratório de Biologia Molecular e Doenças Endêmicas. Instituto Oswaldo Cruz - Fiocruz. Rio de Janeiro, RJ, Brasil; Laboratório de Pesquisa em Leishmaniose. Instituto Oswaldo Cruz - Fiocruz. Rio de Janeiro, RJ, Brasil; Laboratório de Biologia Molecular e Doenças Endêmicas. Instituto Oswaldo Cruz - Fiocruz. Rio de Janeiro, RJ, Brasil; Laboratório de Doenças Parasitárias. Instituto Oswaldo Cruz - Fiocruz. Rio de Janeiro, RJ, Brasil; Laboratório de Biologia Molecular e Doenças Endêmicas. Instituto Oswaldo Cruz - Fiocruz. Rio de Janeiro, RJ, Brasil

Comments about the finding of *Trichophoromyia auraensis* (Mangabeira 1942) sandfly specimens infected by *Leishmania* ( *Viannia* ) parasites in Acre state

Since 2011, our group has been conducting field studies in the state of Acre, trying to contribute with new findings about phlebotomine sandflies fauna and potential leishmaniasis vectors in the state. In an epidemiological context, Amazon is undergoing rapid changes in environmental conditions that directly affect the population of sandfly vectors and therefore disease transmission. Although considered a region with an increasing incidence of cutaneous leishmaniasis, knowledge of the geographical distribution of phlebotomine sandflies associated with disease transmission is still scarce for some regions, such as Acre state, where the cutaneous manifestation of the disease reached high prevalence in the last years ( Martins & Silva 1964 , Arias & Freitas 1982 , Arias et al. 1984 , Silva-Nunes et al. 2008 , Azevedo et al. 2008 , Teles et al. 2013, d , de Araujo-Pereira et al. 2014 , Oliveira et al. 2015 ).

From 2011 to 2012, we centralised the sandfly captures in residential areas within forested regions and recreational areas of Rio Branco municipality, the capital of Acre, and found a very diverse fauna in urban and peri urban areas of the city, as diverse as that encountered in areas of primary forest. A total of 16 Phlebotominae genera and 23 species was taxonomically identified, being *Trichophoromyia auraensis* and *Nyssomyia whitmani* the most abundant species in both forested and peridomiciliary environments. Taking into account the high prevalence of *Th* . *auraensis* in Rio Branco (more than 50% of the total captured insects), we reported in 2014, this species as a putative vector in the region (de Araujo-Pereira et al. 2014 ).

The manuscript by Oliveira et al. (2015) describes *Trichophoromyia* *ruifreitasi* , a new phlebotomine sandfly species from Acre, morphologically similar to *Th* . *auraensis* , that was identified between male specimens collected in the municipality of Assis Brasil ( Oliveira et al. 2015 , Teles et al. 2016 ). As mentioned by Oliveira et al. (2015) , “ *This species is similar to Th. auraensis (Mangabeira)* , *but the two species can be easily differentiated by the distribution of setae on their parameres, and by the presence of a dorsal lobe in the parameres of the new species* ”. In this study, the authors detailed the morphological characteristics of male specimens of *Th* . *ruifreitasi* identified in the municipality of Assis Brasil ( Oliveira et al. 2015 ). In contrast to the investigation by Teles et al. (2016) performed in the municipality of Assis Brasil, in the study areas of Rio Branco, we identified all male specimens of the *Trichophoromyia* genus as *Th* . *auraensis* (de Araujo-Pereira et al. 2014 ). The study by Oliveira et al. (2015) already mention our investigation about the sandfly fauna in Rio Branco, in which we report *Th* . *auraensis* as a putative vector in this Amazonian region (de Araujo-Pereira et al. 2014 ). Valdivia et al. (2012) were the first to identify *Th* . *auraensis* as a natural carrier of *Leishmania* ( *Viannia* ) *lainsoni* and *Leishmania* ( *Viannia* ) *braziliensis* in Madre de Dios, an Amazon region in Peru bordering the state of Acre. Similar to that observed in Rio Branco, in this region of Peru, *Th* . *auraensis* was also the most abundant sandfly species (63%), and the minimum infection prevalence was estimated to be 0.6% by kDNA-polymerase chain reaction (kDNA-PCR) performed in insect pools. Using a FRET-based real time PCR, the authors identified four *Th* . *auraensis* pools positive for *L* . ( *V* .) *lainsoni* and *L* . ( *V* .) *braziliensis* ( Valdivia et al. 2012 ).

Proceeding with our investigation in Rio Branco, we further analysed a sampling of non-blood-fed female sandflies for the presence of *Leishmania* DNA in each insect, individually. The choice for performing the molecular diagnosis separately, by individual specimen instead of pools of insects, was due to the complexity and diversity of the sandfly fauna in the Amazon Basin. Using a multiplex PCR directed simultaneously to *Leishmania* kDNA and to the IVS6 cacophony gene region in neotropical sandflies, coupled to hybridisation with a *L* . ( *Viannia* ) biotinylated probe (de Pita-Pereira et al. 2005 ), total positivity of 6.94% was found in the municipality of Rio Branco. The molecular characterisation of *Leishmania* spp. was performed by sequencing the 234 bp fragment obtained by amplifying a region of the *hsp70* gene from each positive sample, previously assayed for the presence of *Leishmania* kDNA (da Graça et al. 2012 ). *L* . ( *V* .) *braziliensis* DNA was identified in *Th* . *auraensis,* *Evandromyia saulensis* and *Pressatia* sp. individuals. For the other positive samples of *Th* . *auraensis* and *Ev* . *saulensis* , the identification was restricted to the *Viannia* genus. These data were recently published in Memórias do Instituto Oswaldo Cruz (de Araujo-Pereira et al. 2017 ). The finding of *L* . ( *Viannia* ) DNA in two *Ev* . *saulensis* , with the confirmation of *L* . *braziliensis* in one specimen, corresponds to the first record of possible infection associated with this sandfly. Moreover, we registered in Rio Branco, Acre state, *Th* . *auraensis* and *Pressatia* sp. infected by *L* . ( *Viannia* ) parasites (in three out of nine positive *Th* . *auraensis* individuals and in the only positive Pressatia sp., the genetic material was confirmed as *L* . *braziliensis* ). The role of these species as vectors of parasites responsible for New World tegumentary leishmaniasis remains to be established for better understanding the risk of the disease transmission in the Neotropics (de Araujo-Pereira et al. 2017 ).

In our opinion, both studies ( Teles et al. 2016 , de Araujo-Pereira et al. 2017 ) were published in very close periods, almost in parallel. Our manuscript was submitted in June 2016 and the one by the group of Teles was published in July 2016, during the peer-revision process of our manuscript.

Finally, we highlighted differences on some technical and critical aspects between the two studies. In Teles et al. (2016) , the collected sandfly females from Assis Brasil, Acre state, were assembled in groups (2-20 specimens per group), and each pool was composed by the same species, date and place of capture. Because females of *Th* . *auraensis* and *Th* . *ruifreitasi* are probably indistinguishable, the authors named them *Th* . *auraensis* / *Th* . *ruifreitasi* [Lu. *auraensis* /Lu. *ruifreitasi* , according to Young and Duncan (1994)]. Teles et al. (2016) found a minimal infection prevalence of 2.05% in *Lu* . *auraensis* / *Lu* . *ruifreitasi* (16 out of 58 pools) by parasites from the *L* . *braziliensis* complex. If one considers the limitation to morphologically differentiate between sandfly females of both species, our study in Rio Branco was advantageous, since there was no overlap among *Th* . *auraensis* and *Th* . *ruifreitasi* in the study areas. All male specimens of the *Trichophoromyia* genus were previously identified as *Th* . *auraensis* (de Araujo-Pereira et al. 2014 ), and were individually analysed for the presence of *Leishmania* DNA (de Araujo-Pereira et al. 2017 ).

In general, both studies ( Teles et al. 2016 , de Araujo-Pereira et al. 2017 ) are complementary, being performed in two municipalities of the state of Acre with high prevalence of cutaneous disease and thus bringing important contribution in demonstrating the diversity of sandfly species with potential involvement in the cutaneous leishmaniasis transmission cycle in the investigated areas.

## References

[B8] Arias JR, Freitas RA, Barrett TV (1984). A new sandfly in the subgenus Nyssomyia (Diptera, Psychodidae) from the Amazon Basin of Brazil. Mem Inst Oswaldo Cruz.

[B9] Arias JR, Freitas RA (1982). The known geographical distribution of sandflies in the state of Acre, Brazil (Diptera: Psychodidae). Acta Amaz.

[B10] Azevedo ACR, Costa SM, Pinto MCG, Souza JL, Cruz HC, Vidal J (2008). Studies on the sandfly fauna (Diptera: Psychodidae: Phlebotominae) from transmission areas of American cutaneous leishmaniasis in state of Acre, Brazil. Mem Inst Oswaldo Cruz.

[B11] da Graça GC, Volpini AC, Romero GAS, de Oliveira MP, Hueb M, Porrozzi R (2012). Development and validation of PCR-based assays for diagnosis of American cutaneous leishmaniasis and identification of the parasite species. Mem Inst Oswaldo Cruz.

[B12] de Araujo-Pereira T, de Pita-Pereira D, Boité MC, Melo M, da Costa-Rego TA, Fuzari AA (2017). First description of Leishmania (Viannia) infection in Evandromyia saulensis, Pressatia sp. and Trichophoromyia auraensis (Psychodidae: Phlebotominae) in a transmission area of cutaneous leishmaniasis in Acre state, Amazon Basin, Brazil. Mem Inst Oswaldo Cruz.

[B13] de Araujo-Pereira T, Fuzari AA, Andrade JD, de Pita-Pereira D, Britto C, Brazil RP (2014). Sand fly fauna (Diptera: Psychodidae: Phlebotominae) in an area of leishmaniasis transmission in the municipality of Rio Branco, state of Acre, Brazil. Parasit Vectors.

[B14] de Pita-Pereira D, Alves CR, Souza MB, Brazil RP, Bertho A, Barbosa A (2005). Identification of naturally infected Lutzomyia intermedia and Lutzomyia migonei with Leishmania (Viannia) braziliensis in Rio de Janeiro (Brazil) revealed by a PCR multiplex non-isotopic hybridization assay. Trans R Soc Trop Med Hyg.

[B15] Martins AV, Silva JE (1964). Notas sobre os flebotomíneos do estado do Acre, com a descrição de duas espécies novas (Diptera, Psychodidae). Rev Bras Biol.

[B16] Oliveira AFJ, Teles CBG, Medeiros FM, Camargo LMA, Pessoa FAC (2015). Description of Trichophoromyia ruifreitasi, a new phlebotomine species (Diptera, Psychodidae) from Acre state, Brazilian Amazon. Zookeys.

[B17] Silva-Nunes M, Cavasini CE, Silva NS, Galati EAB (2008). Epidemiologia da Leishmaniose Tegumentar e descrição das populações de flebotomíneos no município de Acrelândia, Acre, Brasil. Rev Bras Epidemiol.

[B18] Teles CBG, dos Santos APA, Freitas RA, de Oliveira AFJ, Ogawa GM, Rodrigues MS (2016). Phlebotomine sandfly (Diptera: Psychodidae) diversity and their Leishmania DNA in a hot spot of American Cutaneous Leishmaniasis human cases along the Brazilian border with Peru and Bolivia. Mem Inst Oswaldo Cruz.

[B19] Teles CBG, Freitas RA, Oliveira AFJ, Ogawa GM, Cavalcante EA, Medeiros JF (2013). Description of a new phlebotomine species (Diptera: Psychodidae, Phlebotominae) and new records of sand flies from the state of Acre, Northern Brazil. Zootaxa.

[B20] Valdivia HO, de los Santos MB, Fernández R, Baldeviano GC, Zorrilla VO, Vera H (2012). Natural Leishmania infection of Lutzomyia (Trichophoromyia) auraensis in Madre de Dios, Peru, detected by a fluorescence resonance energy transfer-based real-time polymerase chain reaction. Am J Trop Med Hyg.

[B21] Young DG, Ducan NA (1994). Guide to the identification and geographic distribution of Lutzomyia sandflies in Mexico, the West Indies, Central and South America (Diptera: Psychodidae). Mem Am Entomol Inst.
